# Constitutive expression of the Type VI Secretion System carries no measurable fitness cost in *Vibrio cholerae*

**DOI:** 10.1002/ece3.11081

**Published:** 2024-03-01

**Authors:** Christopher Zhang, Sayantan Datta, William C. Ratcliff, Brian K. Hammer

**Affiliations:** ^1^ School of Biological Sciences Georgia Institute of Technology Atlanta Georgia USA; ^2^ Interdisciplinary Graduate Program in Quantitative Biosciences Georgia Institute of Technology Atlanta Georgia USA

**Keywords:** evolution, fitness cost, microbial community dynamics, microbial warfare, Type VI Secretion System

## Abstract

The Type VI Secretion System (T6SS) is a widespread and highly effective mechanism of microbial warfare; it confers the ability to efficiently kill susceptible cells within close proximity. Due to its large physical size, complexity, and ballistic basis for intoxication, it has widely been assumed to incur significant growth costs in the absence of improved competitive outcomes. In this study, we precisely examine the fitness costs of constitutive T6SS firing in the bacterium *Vibrio cholerae*. We find that, contrary to expectations, constitutive expression of the T6SS has a negligible impact on growth, reducing growth fitness by 0.025 ± 0.5% (95% CI) relative to a T6SS− control. Mathematical modeling of microbial populations demonstrates that, due to clonal interference, constitutive expression of the T6SS will often be neutral, with little impact on evolutionary outcomes. Our findings underscore the importance of precisely measuring the fitness costs of microbial social behaviors and help explain the prevalence of the T6SS across Gram‐negative bacteria.

## Graphical abstract

The T6SS is a bacterial protein secretion system that is many multi‐megadaltons in size, highly complex, and has a very conservative developmental regulation; all prior work has assumed that the T6SS must be metabolically costly to maintain and utilize. In our paper, we use experimental and computational tools from the field of microbial evolution to find that the fitness costs of T6SS expression are negligible. Our paper overturns the widely held belief that the T6SS carries a large intrinsic metabolic cost to utilize.

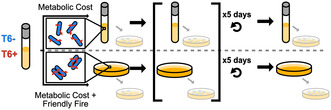

## 1 INTRODUCTION

An important mechanism of intermicrobial antagonism is the Type VI Secretion System (T6SS). The T6SS is a modified phage tail spike that delivers a payload of deadly effector proteins into neighboring competitor bacterial species. Its widespread distribution (~25% of Gram‐negative species; Bingle et al., 2008) can be understood through its outsized effect on microbial dynamics. The T6SS is both potent, being able to deliver toxins with up to a 99.99% fatality rate, and broad‐acting, being able to affect Gram‐negative bacteria, Gram‐positive bacteria, as well as eukaryotic cells (Le et al., 2021; MacIntyre et al., 2010; Pei et al., 2022; Pukatzki et al., 2006). The T6SS is utilized across a broad range of evolutionary niches, facilitating defense against competitors, as well as host invasion through the killing of commensal species (Basler et al., 2013; Chassaing & Cascales, 2018; Drebes Dörr & Blokesch, 2018; García‐Bayona & Comstock, 2018; Smith et al., 2020).

The structure and function of the T6SS has been the subject of intense research since its discovery (Alteri & Mobley, 2016; Basler, 2015; Ho et al., 2014; Nguyen et al., 2018; Unni et al., 2022; Zoued et al., 2014). With the exception of the baseplate and species‐specific effector proteins, crystal structures have been determined for all other proteins comprising the canonical T6SS structure (Nguyen et al., 2018). Studies have also explored the role of T6SS in species where it has been utilized for functions other than antagonism such as ion sequestration, biofilm formation, swarming, as well as various host–microbe interactions (Alteri & Mobley, 2016; Chugani & Greenberg, 2007; Parsons & Heffron, 2005; Shalom et al., 2007).

Given its hypothesized multi‐megadalton size, complexity, and conservative developmental regulation, prior work has inferred that the T6SS must be metabolically costly to maintain and utilize (Alteri & Mobley, 2016; Ho et al., 2014; Smith et al., 2020; Unni et al., 2022). However, recent experiments have not detected a measurable fitness effect of T6SS expression over 24 h of growth in rich media (Robitaille et al., 2023; Septer et al., 2023). Single‐day growth curves have a relatively low sensitivity, however, as they can only measure competitive outcomes over a small number of generations, and are thus ineffective for measuring traits with a small impact on fitness (Yokota & Sterner, 2010). To generate more precise estimates of the fitness costs of constitutive T6SS expression, we examined the outcome of competition between isogenic T6SS− and T6SS+ strains over 5 days (~100 generations) of competition.

## 2 RESULTS

To measure the fitness costs in the T6SS, we used two strains of *Vibrio cholerae* created and validated in Ng et al. (2022) and Thomas et al. (2017) (Table S1), one with constitutive T6SS expression (T6SS+) and a T6SS(−) genotype that includes a transcriptional terminator placed in front of the T6SS operon. We confirmed the identity and killing activity of these engineered strains with killing assays (Figure S1). In addition, we confirmed that the T6SS+ strain expresses high levels of each of the genes in the T6SS operon and the T6SS− strain has abrogated T6SS transcription through RNAseq, consistent with expectations (Figure S2). The difference in growth rate between these two otherwise isogenic strains should therefore be due entirely to the metabolic cost of T6SS protein expression, T6SS assembly, and T6SS firing events. To differentiate these two strains during competition experiments, we also introduced a selectable antibiotic resistance marker for spectinomycin (SpecR) into the lacZ gene of each strain (accounting for the effect of antibiotic resistance via marker‐swap experiments) so that a blue‐white screen can distinguish the two strains.

**FIGURE 1:**
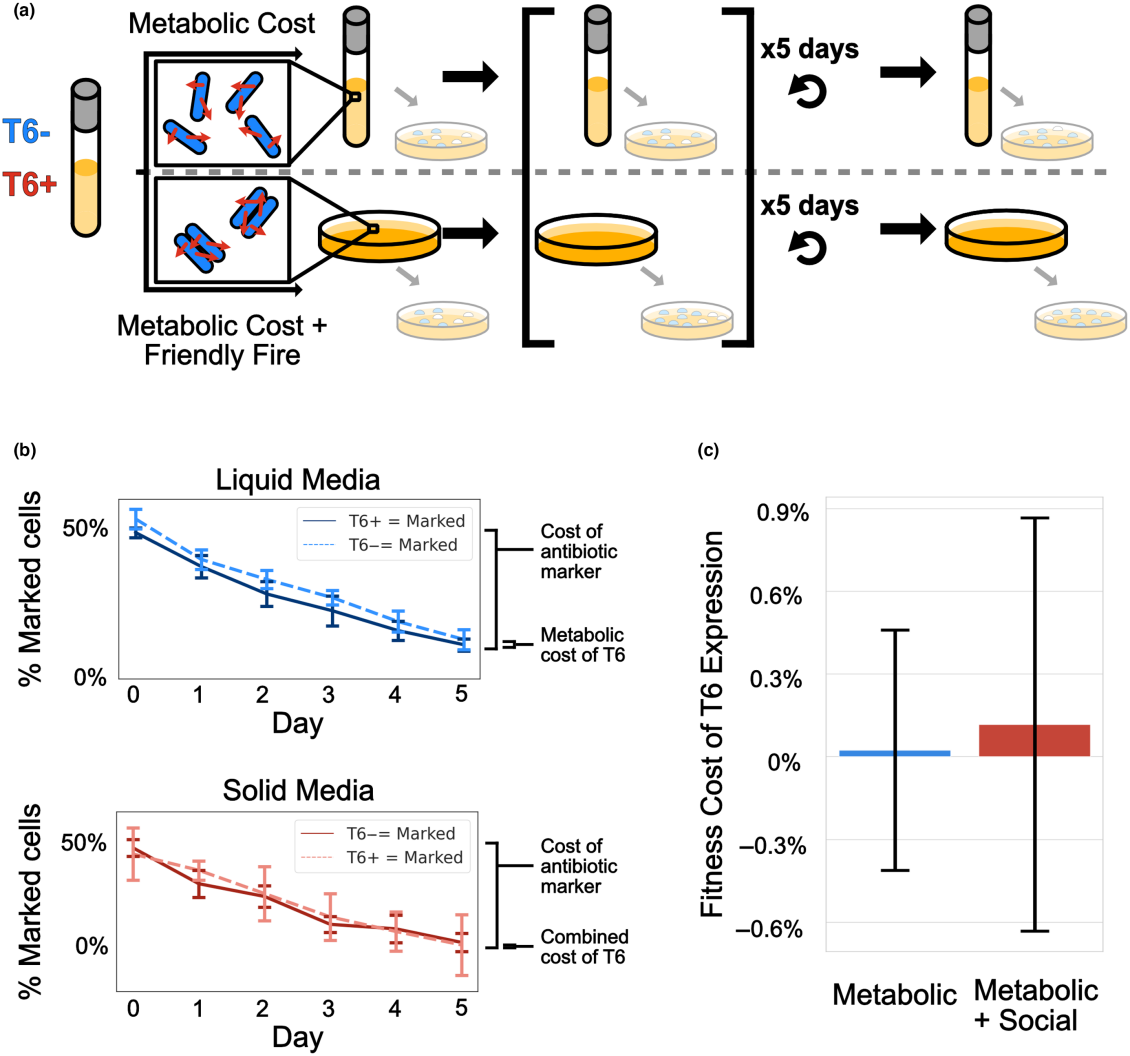
Constitutive expression of the T6SS incurs negligible fitness costs over a 5 day, ~100 generation experiment. (a) Experimental setup for competitions. T6SS+ and T6SS− bacteria were initially mixed in an equal proportion. To measure the metabolic cost of constitutive T6SS expression, the mixture was incubated in liquid culture at 37°C with shaking and back‐diluted daily for five transfers. To measure any additional costs incurred by friendly fire, we performed competitions on LB agar at 37°C, and diluted every 8 h for 5 days. The change in frequency of the T6SS+ strain was measured via aliquoting a portion of the daily transfer for a blue‐white screen. (b) An antibiotic swap experiment shows that the marked cells decrease in each daily transfer for all populations due to the associated costs with the antibiotic marker. The difference in the rate of decrease, which represents the metabolic and combined costs of the T6SS, is minimal. (c) In liquid media, the mean fitness cost of T6SS was 0.025% (nine biological replicates quantified with one CFU plate per replicate, error bars reflect the 95% CI; this value is not significantly different from 0). On solid media, the mean fitness cost of T6SS was 0.11% (six biological replicates quantified with one CFU plate per replicate, error bars reflect the 95% CI; not significantly different from 0).

We measured the fitness costs of T6SS expression under two conditions: in a liquid media, where cells do not directly interact (Speare et al., 2022), and on agar plates (Figure 1a). While both of these conditions include the growth‐rate costs of T6SS expression, growth on solid media also introduces costs associated with friendly fire. Even though clonemates are resistant to the toxins delivered by the T6SS, sublethal toxicity and physical damage to cell walls and plasma membranes may incur an additional cost to T6SS expression when the population is growing as a spatially structured biofilm (Kamal et al., 2020; Liang et al., 2019; McNally et al., 2017). In both cases, we examined competition between T6SS+ and T6SS− strains over ~100 generations. We ran each competition experiment twice, once with the T6SS+/SpecR and once with T6SS−/SpecR. We subtracted the results of the T6SS+/SpecR experiment from the T6SS−/SpecR experiment to isolate only the cost of T6SS (Figure 1b,c).

In liquid media, constitutive T6SS expression reduced fitness by 0.025% ± 0.5% (uncertainty represented as a 95% confidence interval), but this was not statistically distinguishable from zero (*t* = 0.12, *p* = .91, df = 16). On solid media, which includes costs of friendly fire in addition to the metabolic burden of T6SS expression, the effect was similarly small: 0.11% ± 0.75% (95% CI, *t* = 0.35, *p* = .73, df = 9) (Figure 1b,c). For comparison, the cost of the antibiotic resistance, also measured in this experiment, was 2.8% ± 0.2% (95% CI, *t* = 4.24, *p* = .0003, df = 16) (Table S1). In our experiment, T6SS+ cells did not grow at a significantly different rate from T6SS− competitors. However, it is possible the true metabolic cost of T6SS was up to 0.5% (the 95% confidence interval of our liquid media experiments ranged from −0.5% to 0.5%).

**FIGURE 2:**
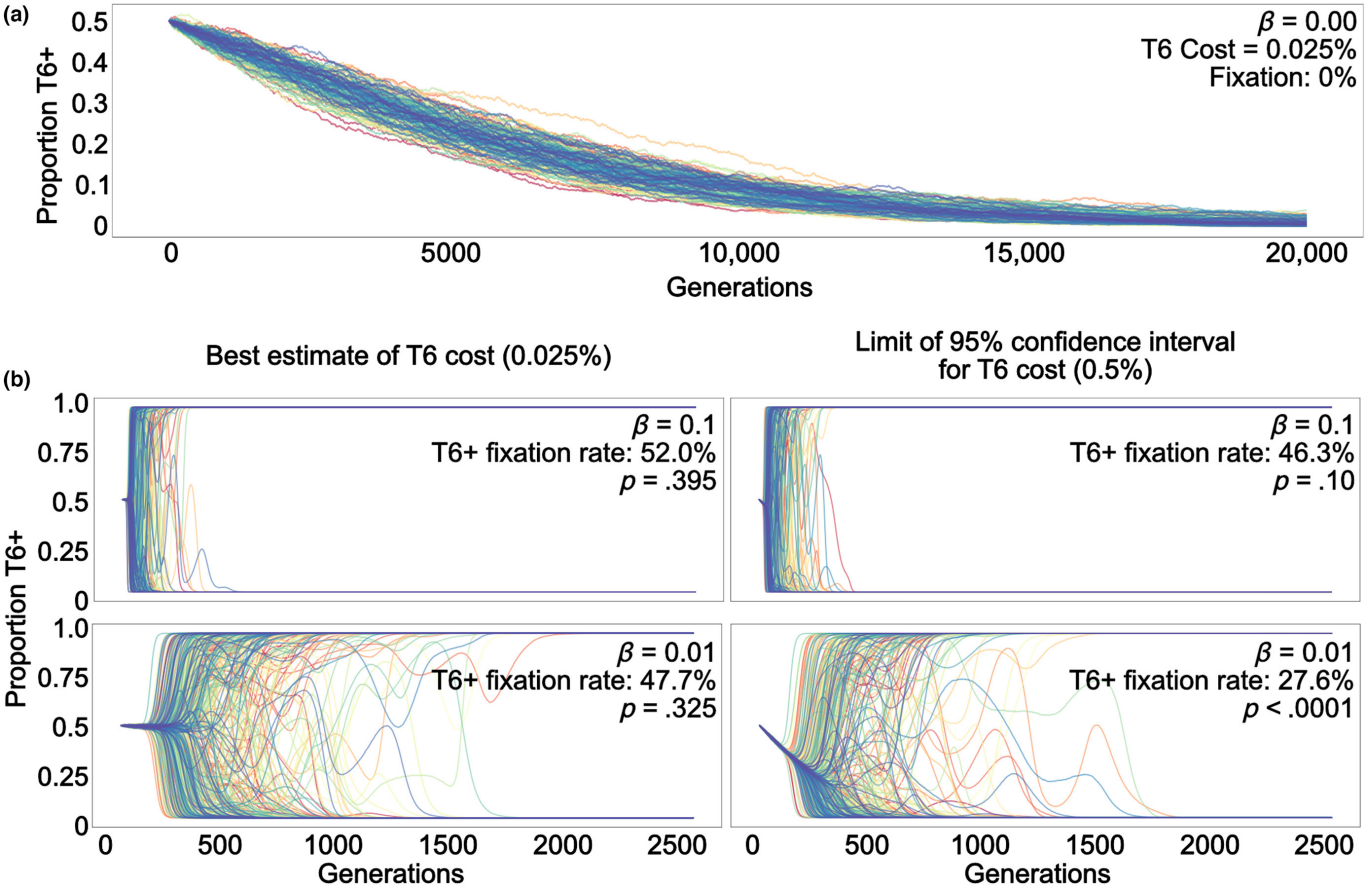
Under most population genetic conditions, clonal interference renders the minor costs of T6 expression evolutionarily neutral. (a, b) Simulations of 1000 bacterial populations consisting of 1 million bacteria using a Wright–Fisher model. (a) In the absence of clonal interference from beneficial mutations (*β* = 0), all T6+ genotypes take more than 20,000 generations to go extinct. (b) Adding novel mutations to the model (*β* > 0), which generates clonal interference, reduces the efficacy of selection against T6 expression. When we use our best estimate of the cost of T6 expression from our experiments (Figure 1, results from liquid media), T6 expression is effectively neutral, even with 10‐fold variation *β*. When the fitness cost of T6 is 20 times higher than our experimental estimate (0.5%), T6 is still neutral when the rate of beneficial mutations arising is moderately high (*β* = 0.1), but not when *β* is lower (0.01).

We can contextualize these costs via simple mathematical models. Assuming the metabolic cost we measured of T6SS (0.025%) is true, a T6SS+ strain starting at 50% frequency in a population of a million bacteria would take more than 20,000 generations to go extinct (Figure 2a; Appendix S1). Real microbial populations are more dynamic, however, as other mutations arise within these lineages and themselves become subject to selection. In asexual organisms like bacteria, this drives a phenomenon known as clonal interference: competition between independent lineages in the population bearing different beneficial mutations. Depending on key population genetic parameters (e.g., the rate and magnitude of beneficial mutations, population size, etc.), mildly beneficial mutations that otherwise would have been fixed may be rendered effectively neutral due to clonal interference (Park & Krug, 2007).

To explore the effects of clonal interference on selection against the T6SS, we created a stochastic Wright–Fisher model that examines the growth and competition of a population of T6SS+ and T6SS− bacteria subject to mutation and selection. We simulated a population of 1 million bacteria, starting with an even ratio of T6SS+ and T6SS− cells. We utilized the parameters from Good et al. (2012). Namely *β*, the magnitude of beneficial mutations, is 0.01; and *m*, the mutation rate per bacteria per generation, is 10^−5^. By varying the supply of beneficial mutations (*β*, larger values result in more clonal interference) as well as the cost of the T6SS, we examine the conditions under which the costs of T6SS are non‐neutral.

Using our best estimate of the metabolic cost of constitutive T6SS expression (0.025%), we find that the T6SS has no effect on evolutionary outcomes at either *β* value simulated (Figure 2b, left), suggesting that at the measured cost, even slow rates of adaptive evolution render the T6SS neutral. However, if we take the worst‐case scenario, and assume that the true cost of the T6SS is 0.5% (the limit of our 95% confidence interval), then the cost of the T6SS is non‐neutral when the supply of novel beneficial mutations is low (*β* = 0.01), but not when it is high (*β* = 0.1, Figure 2b, right). Taken together, these results demonstrate that the T6SS is likely neutral under most population genetic conditions: the only time T6SS cost significantly alters the course of evolution in our simulations is either when T6SS has a statistically improbable cost of expression or when the bacteria have a very low supply of novel beneficial mutations (as might be the case if they were near a fitness optima).

## 3 DISCUSSION

Our study provides novel empirical evidence that constitutive expression of the Type VI Secretion System (T6SS) in *Vibrio cholerae* carries little intrinsic fitness cost. Using precise competition experiments between T6SS+ and T6SS− strains, we demonstrate mean fitness reductions of just 0.025% in liquid and 0.11% on agar plates over ~100 generations of growth (Table S1), though these differences were not significantly different from zero. While our primary goal for comparing liquid vs. solid media was to assess the fitness costs of friendly fire, we acknowledge that these environments will almost certainly incur other differences in growth. Still, our experimental approach controls for environmental effects: by comparing otherwise isogenic T6SS+ and T6SS− cells within each environment, we can isolate just the fitness differences that arise as a consequence of T6SS expression. Our mathematical modeling further illustrates that these fitness costs will often be rendered selectively neutral through clonal interference. The negligible cost of T6SS expression, even under conditions engineered to detect the cost of constitutive expression, may help explain its prevalence across diverse Gram‐negative lineages.

While our results show that the constitutive expression of T6SS by a single strain of *Vibrio cholerae* is not costly under our laboratory conditions (i.e., exponential growth in rich media), this does not mean there will never be a cost for expression. The cost of T6SS expression may be exacerbated under certain conditions, such as growth in oligotrophic conditions. Alternatively, T6SS expression may incur environmentally dependent costs independent of metabolism. For example, recent work has found that the T6SS is strongly selected against within animal models (Robitaille et al., 2023; Septer et al., 2023), though further work will be required to determine the precise mechanistic cause. Taken together with our findings, these results suggest that while the expression of the T6SS is not inherently metabolically costly, it may incur context‐dependent ecological costs (e.g., through triggering host immune response (Gupta et al., 2021; Ma & Mekalanos, 2010), or via emergent consequences of changed ecological dynamics (Robitaille et al., 2023)). Indeed, examining the evolution of the T6SS during adaptation to novel conditions is a broadly promising approach for examining its costs and benefits under diverse real‐world conditions.

This work underscores a central challenge in microbial ecology and evolution: measuring fitness directly is difficult, time‐consuming, and ecologically context‐dependent. In contrast, fitness proxies (e.g., the ATP consumption of a given trait) may be relatively easy to estimate from first‐principles or existing datasets. Yet if fitness proxies have little predictive value in experiments directly measuring fitness, then this highlights the crucial need for direct experimental measurement. Multi‐transfer competition experiments are a powerful and greatly under‐utilized approach for examining even small differences in fitness. In contrast to standard methods, like a 24‐h growth curve, competition experiments amplify the effects of small competitive differences across a greater number of generations, effectively increasing the signal (selection) to noise (sampling and measurement error) ratio of the experiment (Yokota & Sterner, 2010).

The T6SS is just one example of a sophisticated and energetically expensive microbial weapon whose expression is widely assumed to be costly. For example, the Type III and Type IV Secretion Systems are also large membrane‐spanning multiprotein complexes consisting of large proteins that are under tight genetic control and require a large amount of ATP (Ilangovan et al., 2015; San Millan & MacLean, 2017; Sturm et al., 2011; Zoued et al., 2014). Future work examining the direct fitness costs of these traits under controlled laboratory conditions would be illuminating.

Our study challenges the widespread assumption that expression of the T6SS in bacteria imposes a significant metabolic burden, reducing a clonal lineage's growth rate (and thus its fitness during growth). We show that even constitutive expression of the T6SS in *Vibrio cholerae* has a negligible impact on fitness during growth. This finding may help account for the widespread distribution of the T6SS among Gram‐negative bacteria, as it can confer a large competitive advantage against other bacteria at minimal cost. However, we caution that the fitness consequences of T6SS expression may depend on ecological and genetic factors that warrant further investigation. Our study also highlights the importance of precise fitness measurements when evaluating microbial social traits, and demonstrates how modest behavioral costs can be rendered neutral by clonal interference.

## 4 METHODS

### 4.1 Bacterial strains and media

Bacterial strains were grown aerobically at 37°C overnight in lysogeny broth (LB) (1% w/v tryptone (Teknova, Hollister, CA, USA), 0.5% w/v yeast extract (Hardy Diagnostics, Santa Maria, CA, USA), 1% w/v NaCl (VWR Life Sciences, Radnor, PA, USA)) with constant shaking or on LB agar (1.5% w/v agar; Genesee Scientific, San Diego, CA, USA) standing at 37°C. LB‐X‐gal was made by mixing in 40 μg/mL of X‐gal (GoldBio, St. Louis, MO, USA) to LB agar while it is liquid.

### 4.2 Mutant construction

All *V. cholerae* mutant strains were made using the pKAS allelic exchange system described by Skorupski et al. using pKAS32 (Skorupski & Taylor, 1996). JT101 and SN598 (Table S1) were described in previous studies (Ng et al., 2022; Thomas et al., 2017). CZ005 and CZ006 were generated through an insertion of a spectinomycin resistance cassette into the lacZ gene of JT101 and SN598, respectively. All insertions and changes to phenotype were confirmed with PCR, antibiotic screening, and killing assays.

### 4.3 Liquid LB competition experiment

Overnight cultures of *V. cholerae* were normalized to an OD600 = 1, then mixed in a 1:1 ratio by volume. Each mixture was then serially diluted and 100 μL of the 10^−3^ dilution was mixed into 5 mL of LB and incubated overnight shaking at 37°C. Hundred microliter of the 10^−5^ dilution was plated onto an LB X‐gal plate for quantification with blue‐white screening. For the subsequent 4 days, each overnight mixture was serially diluted and 100 μL of the 10^−5^ dilution was mixed into 5 mL of LB and the 10^−6^ dilution was plated onto an LB X‐gal plate for quantification. Fitness was calculated by finding the ratio of Malthusian parameters as described in Lenski et al. (1991).

### 4.4 Solid agar competition experiment

Overnight cultures of *V. cholerae* were normalized to an OD600 = 1, then mixed in a 1:1 ratio by volume. Each mixture was then serially diluted and 100 μL of the 10^−5^ dilution was plated onto LB X‐gal as well as on LB. The X‐gal plate was saved for quantification. The LB plate was incubated standing at 37°C. Every following 8 h for 5 days, the LB plate was taken out of the standing incubator and all of the agar was scraped off of the plate and transferred into a 50 mL conical tube containing 10 mL of LB. This conical tube was vortexed for 30 s and 300 μL of the supernatant was transferred into a 96‐well plate and serially diluted. Hundred microliter that contained approximately 200–1000 CFU per 100 μL was transferred onto an LB plate for the next time point. Every three time points were recorded via blue‐white screening by plating 100 μL of the serial dilution that contained between 30 and 300 CFU per 100 μL onto X‐gal for quantification. Fitness was calculated with the same calculation as the Liquid LB competition experiment.

### 4.5 Wright–Fisher model

The Wright–Fisher model is a standard framework used in evolutionary biology to describe stochastic allelic evolutionary processes including genetic drift, and in our paper, clonal interference. In a Wright–Fisher simulation, discrete generations of bacteria are calculated by drawing from a weighted binomial distribution from the previous generation. To simulate the effects of clonal interference, each cell had a 10^−5^ chance of mutation every generation. When a cell was mutated, the overall fitness effect of the mutation was drawn from an exponential distribution with the parameter *β* derived from Good et al. (2012). Each simulation was run 1000 times and 200 random runs were plotted for clarity.

### 4.6 Statistics

The 95% confidence interval for both competition assays was calculated with a two sample *t*‐test for the difference in two means. See Tables [Link], [Link] for details. The *p* values in Figure 2 were calculated by counting the quantity of populations that reached fixation or extinction after 5000 simulated generations and using a *χ* ^2^‐test to test the null hypothesis that the T6SS+ strain will go to fixation 50% of the time.

### 4.7 Killing assay

Overnight cultures of *V. cholerae* and *E. coli* (resistant to chloramphenicol) were normalized to an OD600 = 1, then mixed in a 10:1 ratio of *V. cholerae* to *E. coli* by volume. Five microliter of this mixture was spotted onto an LB agarose plate and left to dry for 10 min. The plate was then incubated at 37°C for 3 h. The spot on the plate was cut out and vortexed with 5 mL of LB broth for 30 s. The broth was then serially diluted and 5 μL of each of the serial dilution mixtures were spotted onto an LB chloramphenicol plate.

### 4.8 RNA sequencing and analysis

Overnight cultures of T6SS+, T6SS−, and WT *Vibrio cholerae* were grown at 37°C in lysogeny broth (LB) (*n* = 2 biological replicates). RNA was extracted from these cultures using an Qiagen RNeasy mini kit (Qiagen, Hilden, Germany). An RNA library was prepared using the NEB Ultra II directional RNA library prep kit (New England Biolabs, Ipswitch, MA, USA) and the QIASeq Fast select ‐RNA HMR kit (Qiagen, Hilden, Germany) was used for bacterial rRNA depletion. The completed bacterial RNA library was sequenced on the Illumina NovaSeq 6000 (Illumina, San Diego, CA, USA). To analyze the transcriptomic data, we first trimmed adapters and filtered low‐quality reads using Trimmonatic (v0.39) (Bolger et al., 2014). We then perform alignment of paired reads to reference contigs of *V. cholerae* C6706 (NCBI RefSeq assembly GCF_009763945.1) using STAR (v 2.7.11a) (Dobin et al., 2013) to create binary alignment files (BAM) sorted by genomic coordinates. We counted aligned fragments to all annotated loci in NCBI Refseq annotation using featureCounts (v2.0.6) (Liao et al., 2014). Fragment counts were filtered for low expression and used for differential expression analysis using DESeq2 in R (Love et al., 2014).

## AUTHOR CONTRIBUTIONS

**Christopher Zhang:** Conceptualization (lead); data curation (lead); formal analysis (lead); investigation (lead); methodology (lead); software (lead); validation (lead); visualization (lead); writing – original draft (lead); writing – review and editing (lead). **Sayantan Datta:** Data curation (supporting); formal analysis (supporting); methodology (supporting); software (supporting). **William C. Ratcliff:** Conceptualization (equal); funding acquisition (lead); investigation (equal); methodology (equal); supervision (equal); writing – review and editing (supporting). **Brian K. Hammer:** Conceptualization (equal); funding acquisition (lead); investigation (equal); methodology (equal); resources (lead); supervision (equal); writing – review and editing (supporting).

## CONFLICT OF INTEREST STATEMENT

The authors declare no conflict of interests.

## Supporting information


Appendix S1

Figure S1

Figure S2

Table S1

Table S2

Table S3

Table S4

Table S5

Table S6

Table S7

Table S8

Table S9

## Data Availability

The data underlying this article are available in the article and in its online supplementary material. All supplementary files may be accessed through dryad before publication through the following link: https://datadryad.org/stash/share/QAthLANcMtpm2v_QOm9xc_xLCUoMYyL1uj85cq0QvO4. Supplementary files may be accessed through dryad after publication through the following link: https://doi.org/10.5061/dryad.9cnp5hqqc. RNA sequencing data will be submitted through NCBI and will be available before publication.
