# Supplementary figures and images for: Constitutive expression of the Type VI Secretion System carries no measurable fitness cost in Vibrio cholerae
Source: Ecol Evol. 2024 Mar 1;14(3):e11081. doi: 10.1002/ece3.11081 (PMC10905242; doi:10.1002/ece3.11081)

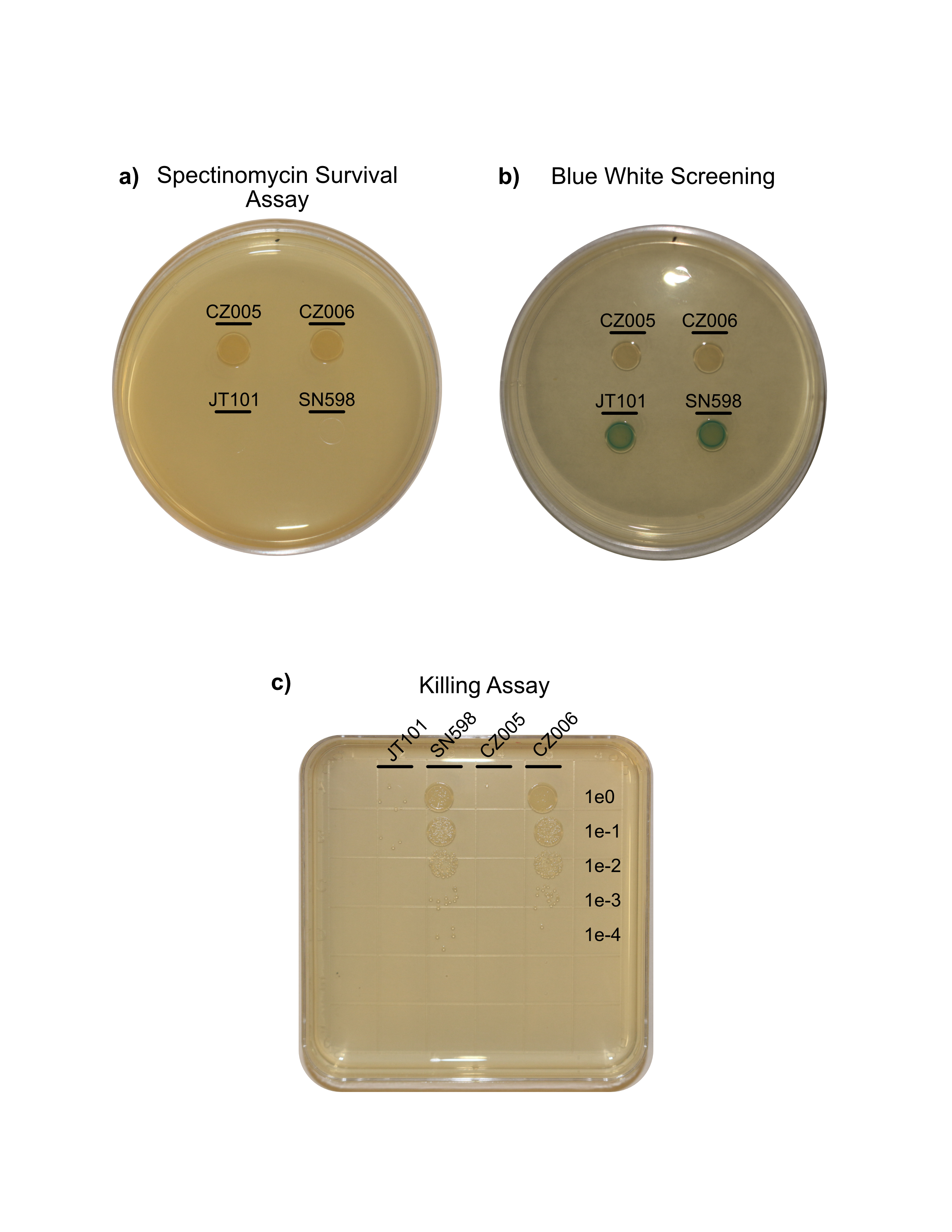

Supplement: Supplementary file 2 — Figure S1 [file ECE3-14-e11081-s006.tiff]

a.

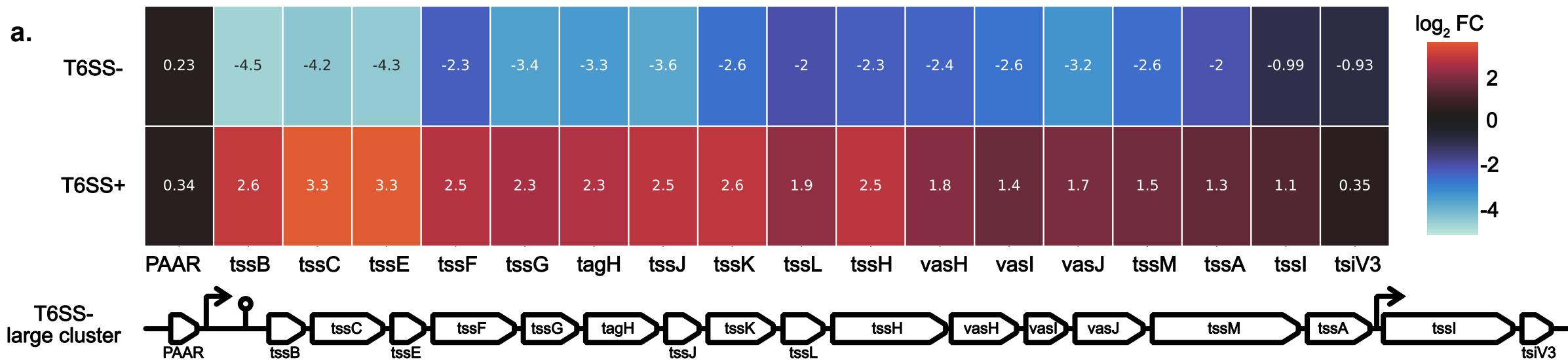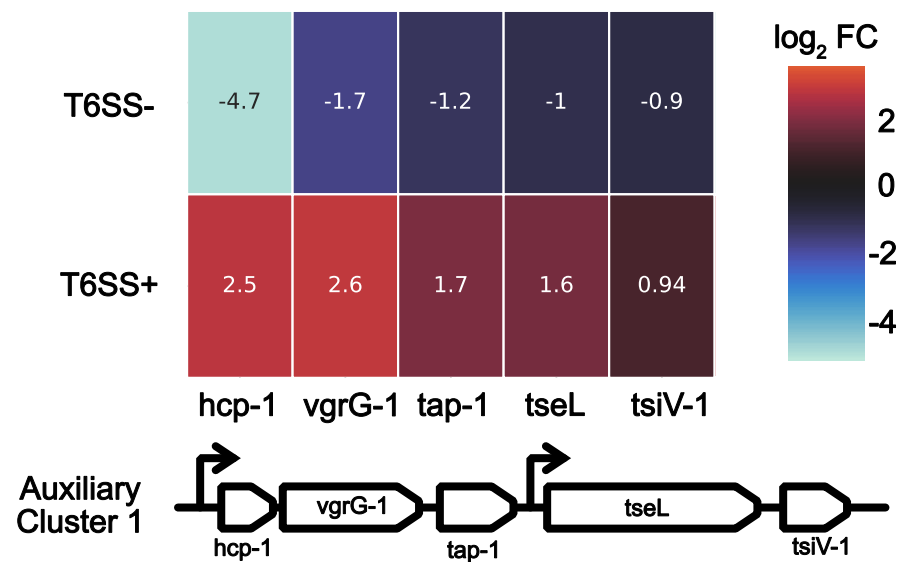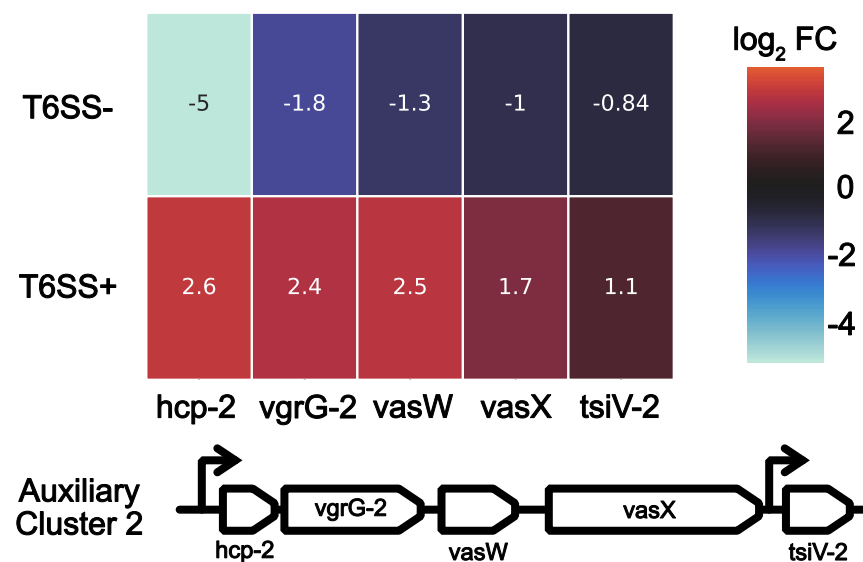

Supplement: Supplementary file 3 — Figure S2 [file ECE3-14-e11081-s008.pdf]
